# Extended Grammar of Systematized Nomenclature of Medicine – Clinical Terms for Semantic Representation of Clinical Data: Methodological Study

**DOI:** 10.2196/80314

**Published:** 2026-01-28

**Authors:** Christophe Gaudet-Blavignac, Julien Ehrsam, Monika Baumann, Adel Bensahla, Mirjam Mattei, Yuanyuan Zheng, Christian Lovis

**Affiliations:** 1 Division of medical information sciences Diagnostic department University Hospital of Geneva Geneva Switzerland; 2 Department of radiology and medical informatics Faculty of medicine University of Geneva Geneva Switzerland

**Keywords:** data models, formal grammar, knowledge representation, semantic interoperability, terminology standards

## Abstract

**Background:**

Interoperability has been a challenge for half a century. Led by an informatics view of the world, the quest for interoperability has evolved from typing and categorizing data to building increasingly complex models. In parallel with the development of these models, the field of terminologies and ontologies emerged to refine granularity and introduce notions of hierarchy. Clinical data models and terminology systems vary in purpose, and their fixed categories shape and constrain representation, which inevitably leads to information loss.

**Objective:**

Despite these efforts, semantic interoperability remains imperfect. Achieving it is essential for effective data reuse but requires more than rich terminologies and standardized models. This methodological study explores the extent to which the SNOMED CT (Systematized Nomenclature of Medicine – Clinical Terms) compositional grammar can be leveraged and extended to approximate a formal descriptive grammar, allowing clinical reality to be expressed in coherent, meaningful sentences rather than preconstrained categories.

**Methods:**

Building on a decade of semantic representation efforts at the Geneva University Hospitals, we developed a framework to identify recurring semantic gaps in clinical data. We addressed these gaps by systematically modifying the SNOMED CT Machine Read` Concept Model and extending its Augmented Backus-Naur Form syntax to support necessary grammatical structures and external vocabularies.

**Results:**

This approach enabled the semantic representation of over 119,000 distinct data elements covering 13 billion instances. By extending the grammar, we successfully addressed critical limitations such as negation, scalar values, uncertainty, temporality, and the integration of external terminologies like Pango. The extensions proved essential for capturing complex clinical nuances that standard precoordinated concepts could not represent.

**Conclusions:**

Rather than creating a new standard from scratch, extending the grammatical capabilities of SNOMED CT offers a viable pathway toward high-fidelity semantic representation. This work serves as a proof-of-concept that separating the rules of composition from vocabulary allows for a more flexible and robust description of clinical reality, provided that challenges regarding governance and machine readability are addressed.

## Introduction

### Status of Health Care Interoperability

Achieving semantic interoperability remains a central challenge in biomedical informatics and health data integration. While technical interoperability ensures that systems can exchange data in compatible formats, semantic interoperability guarantees that exchanged information is unambiguously interpretable across heterogeneous systems and contexts. This objective requires the explicit representation of meaning through formal models, ontologies, and standardized vocabularies, enabling computational reasoning and automated integration of clinical data.

Significant efforts have been invested in developing tools and frameworks to bring semantics to the complex field of health data. In Switzerland, the Swiss Personalized Health Network and its 3-pillar strategy defined a strong semantic representation of data as the first and mandatory pillar [1]. At the European level, the European Health Data Space initiative has further emphasized the importance of standardized, interoperable health data to enable secure sharing and secondary use across member states [2]. Similarly, in the United States, the Office of the National Coordinator for Health IT promotes this goal through the US Core Data for Interoperability, a standardized set of data elements required for nationwide health information exchange [3]. For years, the field of semantic interoperability has been supported by clinical data models, considered technical standards, and classifications and terminologies, called semantic standards.

The multiplication of semantic standards has prompted the need for ontology alignment, or ontology matching, to establish semantic correspondences between heterogeneous terminologies and domain ontologies. Classical systems such as AgreementMaker leverage a combination of lexical similarity, structural consistency, and description logic reasoning to ensure coherent mappings between ontologies [4]. More recent approaches, such as BERTMap, apply contextual language models to improve recall and precision in complex clinical terminologies [5].

Compositional grammar (CG) models provide a structured means of representing the internal semantics of clinical expressions. Clinical concepts are inherently compositional and thus require formalisms that can represent their components and relationships. Frameworks such as the SNOMED CT (Systematized Nomenclature of Medicine – Clinical Terms) CG define syntactic and semantic rules for postcoordinated expressions (PCEs), ensuring internal consistency and machine interpretability [6].

Semantic interoperability also relies on formal logic–based models, typically expressed in Description Logics (DL), which underpin the Web Ontology Language. These models enable key reasoning tasks such as classification, consistency checking, and inferencing [7]. Building on this foundation, knowledge graphs have emerged as a flexible paradigm for integrating heterogeneous biomedical data, representing entities and their relationships within a unified semantic space.

Several international standardization bodies have developed complementary frameworks to support semantic interoperability. Health Level Seven Fast Healthcare Interoperability Resources (FHIR) provides a resource-oriented model for data exchange, including semantic bindings to standard terminologies such as SNOMED CT, Logical Observation Identifiers Names and Codes (LOINC), and Unified Code for Units of Measure, and offers Resource Description Framework and Web Ontology Language representations for semantic web integration [8]. The Observational Medical Outcomes Partnership (OMOP) Common Data Model, developed within the Observational Health Data Sciences and Informatics initiative, harmonizes observational health data using standardized vocabularies and facilitates analytical interoperability [9]. Cross-domain efforts, including ISO (International Organization for Standardization) 23903 and the World Wide Web Consortium’s Health Care and Life Sciences group, promote semantic harmonization through formal metadata and linked data principles [10].

**Figure 1:**
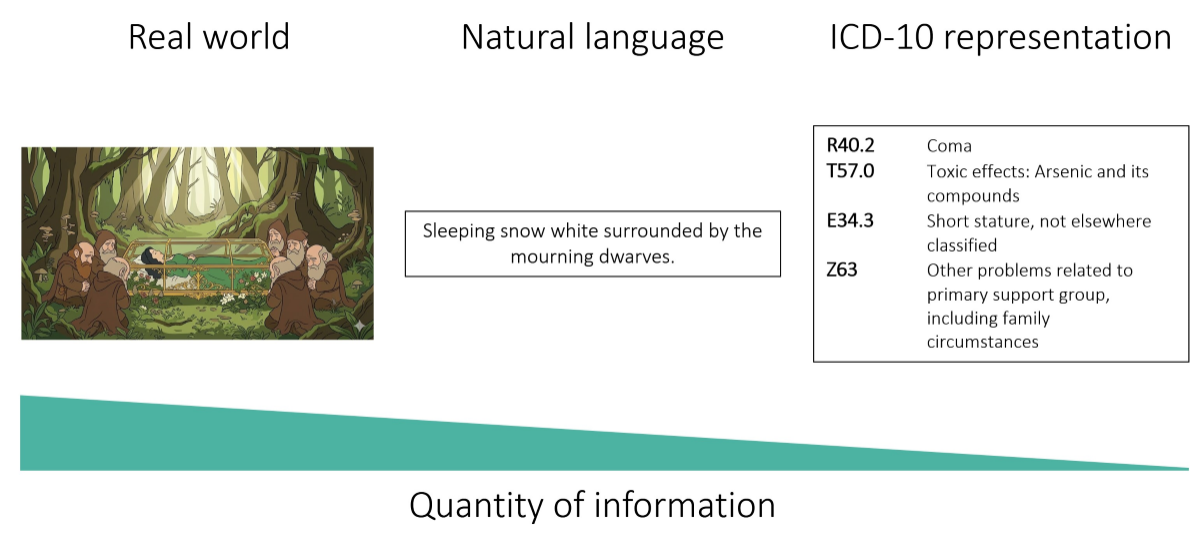
Information loss when using single standards to represent reality.

Each of these models has its strengths and weaknesses, and no system fits all purposes [11-13]. Creating a data model ultimately involves defining a finite set of categories, making choices that inherently shape what can and cannot be represented. These reflect the intended purpose of the model, but fitting data into these finite sets inevitably modifies it, making reuse based on model-specific categories less granular [14]. Semantic standards similarly have specific purposes, and granularity can vary dramatically depending on the focus of the classification. The *ICD-10* (*International Statistical Classification of Diseases, Tenth Revision*) can be useful in representing disease, but cannot represent surgical interventions. Representing data using only single codes from a classification will inherently result in information loss that will be tied to the focus and granularity of the terminology. Figure 1 depicts this loss using a commonly known situation and its representation in natural language, then in *ICD-10* codes.

### SNOMED CT and Natural Language

In this landscape, the CG of SNOMED CT, combined with its broad coverage, makes it a strong candidate for semantic interoperability [15]. With its knowledge graph–like structure, it allows intelligent code retrieval through its expression constraint language and gives users the ability to create PCEs to represent absent concepts. This relies on 2 elements. The CG, an Augmented Backus-Naur Form (ABNF) syntax, is used to create composed expressions that can be parsed and evaluated automatically [16]. The Machine Readable Concept Model (MRCM) defines which concepts (domain) can be refined with which relation (attribute) to which values (range). Combining these resources with the editorial guide, which defines attributes-naming conventions, ensures that new expressions comply with SNOMED CT’s rules and grammar [17].

However, while designed for postcoordination and full expressivity of clinical concepts, it is rarely used to its full capacity and is intended for SNOMED CT concepts only [18,19]. It lacks granularity in some fields, such as strains of SARS-CoV-2, while others, such as genomic sequences, are not covered at all. Using SNOMED CT to represent clinical structured data within its expected parameters is eventually restrictive and insufficient to cover everything. Each attribute has a very specific range, domain, definitions, and permitted use cases, known as domain-specific modeling, and extending it eventually becomes necessary [20].

Like natural languages, SNOMED CT can be described as consisting of 3 fundamental components: a vocabulary of words, a grammar defining the rules for their combination, and the resulting sentences. Natural languages, however, allow the creation of any sentence respecting their grammar, regardless of their meaning, and regularly integrate words from other languages. A sentence such as “The ‘entrepreneur’ dropped his children off at ‘kindergarten’ before going to a ‘karaoke’” uses words from French, German, and Japanese, which have been fully integrated into the English language. It is possible to draw inspiration from this structure to form a similar, computer-processable language, and applying this philosophy to SNOMED CT could improve its usability to fully represent clinical data semantically. The field of medical informatics already possesses rich vocabularies such as SNOMED CT, which serve as the source of machine-readable “words,” each with its own specific strengths and weaknesses. They must then be combined according to well-defined grammar rules to form expressions, for which the SNOMED CT CG provides a comprehensive base.

### Beyond SNOMED CT

Despite long-standing efforts to construct a comprehensive medical terminology, a single complete solution remains elusive, often resulting in the use of fragmented assemblies of various systems [21]. What remains necessary is a unifying set of rules to govern composition.

The solution should be formal, as semantic representation requires clear rules for machine readability that define how building blocks assemble into structured expressions. It must be descriptive rather than prescriptive. While grammars guide expression, they should not restrict what can be said a priori but rather ensure reality can be described clearly and consistently. Importantly, the solution must be dynamic, evolving through continuous iteration. Clinical reality is complex and ambiguous, with new findings emerging faster than they can be formally incorporated. Enabling the construction of meaningful expressions even without predefined concepts allows rapid adaptation to this complexity. To the best of our knowledge, such a formal descriptive grammar (FDG) does not exist yet.

This study proposes a first step in this direction by leveraging SNOMED CT to represent clinical data while ensuring machine readability. We summarize requirements gathered through 10 years of manual semantic representation and describe an implementation based on extending SNOMED CT’s CG, syntax, and MRCM. This approach aims to represent clinical data regardless of source, output terminology, or intended use case. While the results presented reflect the current state, they remain open-ended and extensible.

## Methods

### Overview

This work was developed in parallel to the semantic representation of structured data in the Geneva University Hospitals (HUG) data lake over the past decade. It builds upon the methodological framework introduced in *Semantics in Action: a Guide for Representing Clinical Data Elements* [22] and *Scalar Values in SNOMED CT: a Proposed Extension* [23], which proposes a structured and iterative approach for semantically representing clinical data. Counts of represented metadata elements are derived from the Clinical Data Warehouse without individual data extraction.

### Framework

The guide’s competency-building framework and rule-creation cycle were followed, incorporating both manual expert encoding and consensus-based refinement of representation rules through regular focus groups to define the FDG. The method used was gradually complexified in focus groups as more rules became necessary.

The teaching framework focuses on the use of SNOMED CT and follows a 3-part approach. First, SNOMED International’s (SI) introductory training courses and documentation are taken to familiarize newcomers with the basics of standard-based semantic representation [24]. This allows new team members to start work on a simpler 1-dimensional representation, such as a problem list value set [25]. Next, internal documentation and continued practice give trainees a deeper understanding of real data and local specificities, allowing them to represent more complex data. Finally, more advanced courses, such as the SNOMED CT authoring courses, are taken by long-term core team members [26]. These, along with the practical experience gained by taking part in the training, give the ability to supervise newcomers and to participate in the rule creation cycle described below. New team members are always supervised closely by experienced team members and progressively increase the complexity of their tasks as required. Following this framework ensures that team members participating in the discussions around the rules of representation have a common training and vision.

Over time, complex situations encountered during the process made it clear that SI’s rules were too restrictive to allow for complete representation. Initially, only a few internal unwritten rules were agreed upon, but it quickly became evident that, to avoid losing track of erratically evolving rules and changes, a framework was necessary to harmonize the process and maintain coherence. The rule-creation cycle was developed during weekly focus groups, which bring together the team members who participate in the semantic representation effort. They are made up of a core of 4 team members with heterogeneous professional backgrounds, including health care professionals (medical, nursing, midwifery), more technical training (IT, bioinformatics), and administrative knowledge (billing). These can then be joined by others depending on the team’s setup at that time, such as students, new team members, or other colleagues with an occasional interest in a specific topic. The core members all have proficient experience with semantic representation and specifically with SNOMED CT, and are certified by SI through courses such as the authoring certification. The core of the team represents a small, soft-funded group, and the rest of the group has a high turnover rate. Added to this are limited time and resources, which means that there is a strong need to prioritize how and where said resources are applied. Data collections with high clinical value and a high number of instances, such as laboratory procedures and patient formularies, were tackled first. For the same reasons, work is parallelized as team members are each assigned separate tasks. For these reasons, no formal interannotator agreement studies are carried out.

Instead, specific topics are discussed during focus groups, and only when outcomes are unanimously agreed upon are they validated. Focus group topics consist of questions or complex situations each member has identified, and the rule-creation cycle is applied to each. First, existing guidelines and approaches are thoroughly reviewed and applied if they can solve the issue. If no existing solution is deemed satisfactory, an extension to the current rules is necessary, and is put into practice once consensus is reached through rounds of discussions. It is evaluated both for coherence with regard to previous rules and for clarity and semantic accuracy. Satisfactory outcomes are added to the list of grammar rules, which are revisited until deemed sufficiently tested. As this method applies only to situations for which the current set of rules available through SI or other groups is deemed insufficient or not applicable, they were not formally compared to each other on the same cases.

### Reaching SNOMED CT’s Limits

SNOMED CT was selected as the foundational semantic standard for this representation effort due to its comprehensive set of concepts, its adherence to a formal ABNF syntax, and its robust compositional rules that facilitate precise concept modeling. We prioritize the use of single SNOMED CT concepts first whenever they are sufficient to faithfully represent the data’s content. When single concepts fall short, we use postcoordination, leveraging the constraints defined by the MRCM to its full capacity, constructing complex clinical expressions.

If neither approach can adequately capture the semantic content, extending it becomes inevitable as a representation gap is formally identified. This is where SNOMED CT’s inherent extensibility is crucial for addressing domain-specific requirements. To resolve such gaps, we systematically explore possibilities for modifying the underlying grammar. The primary strategy involves altering existing MRCM rules, specifically through domain or range extensions, as referenced in [27]. Should this be insufficient, we analyze the need to include new SNOMED CT attributes within the representation framework. Finally, if essential semantic concepts are entirely absent from SNOMED CT, an external terminology is integrated, which consequently requires corresponding modifications to the ABNF syntax governing the semantic expressions.

The results detail the principal extensions implemented, which enable semantic coverage for a large percentage of the structured data within the HUG’s data lake. The modified SNOMED CT ABNF syntax and MRCM collectively form the contribution of this paper and are available in Multimedia Appendices 1 and 2.

### Ethical Considerations

Access to the HUG database is granted on an individual basis by the institution’s Chief Data Officer and Medical Director and is reviewed and renewed every 6 months. This authorization permits access for the purpose of viewing and extracting aggregated metadata. No individual-level patient data are ever extracted, processed, or analyzed. All results presented in the manuscript consist exclusively of irreversibly aggregated counts derived from metadata and do not allow identification or reidentification of individuals. Consequently, this work does not constitute research involving human beings or health-related personal data within the meaning of the Swiss Human Research Act (SR 810.30) and falls outside its scope [28]. In accordance with the Swiss Human Research Act and the Swiss Federal Act on Data Protection, neither ethics committee approval nor patient consent was required; therefore, no waiver was sought from the cantonal or hospital ethics committee [29].

## Results

### Overview

Several problematic situations were encountered, which required progressively drifting away from SNOMED CT’s official rules. These are common properties or patterns that occur across all or most natural languages, known as linguistic universals, a concept derived from Chomsky’s universal grammar [30]. They are encountered often while representing data, and most can be addressed only partly by applying SNOMED CT’s rules, grammar, and concepts. These gaps in the capacity to represent reality include negation, numeration, or scalar values, and displacement, which includes uncertainty and temporality. Most are included in some way in SNOMED CT’s design, although rarely implemented in practice, and are therefore solvable by expanding the CG. This is done with domain and range extensions, and the addition of new attribute rules [31], which allow better representation.

Other situations, however, are not intended by SNOMED CT and necessitate breaking the mold to make the CG more inclusive. This is done by integrating other terminologies to fill gaps within SNOMED CT that cannot be handled even with postcoordination, such as SARS-CoV-2 strains. The different situations encountered and solutions devised are detailed below with an example for each. The complete list of MRCM modifications is available in Multimedia Appendix 1, and the modifications done for each property are detailed in Textbox 1.

**Modifications done for each property.**

**Negation**

- 45169001 |Without (attribute)|
- 5185003 |Except for (attribute)|
- 408729009 |Finding context (attribute)|
- 408730004 |Procedure context (attribute)|

**Scalars**

- 103373006 |With size (attribute)|
- 410671006 |Date (attribute)|
- 79409006 |Resulting in (attribute)|
- 246205007 |Quantity (attribute)|
- 246262008 |Score (attribute)|

**Uncertainty**

- 408729009 |Finding context (attribute)|
- 408730004 |Procedure context (attribute)|

**Temporality**

- 255234002 |After (attribute)|
- 288556008 |Before (attribute)|
- 371881003 |During (attribute)|
- 103335007 |Duration (attribute)|

### Scalar Values

Scalar values are physical quantities that can be described by a single pure number accompanied by a unit of measurement [32]. Scalars are one of the first domains encountered that were not present in sufficient detail in SNOMED CT. Scalars appear in myriad places in clinical data, whether as laboratory results, scores, sizes, or measurements, and hold great importance for accurate representation of clinical reality [8,18]. In SNOMED CT, integers were previously included as concepts, which have since been removed and replaced by Unicode text expressions preceded by a hash [33]. There remain concepts that contain numerical values in labels, such as quantitative result cutoffs, but not in any attribute relationship, as they are not fully defined [34].

**Figure 2:**
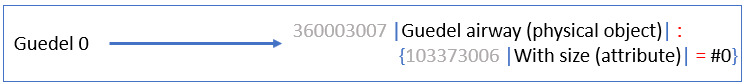
Scalar value representation example.

A better representation of scalar values is achieved with new attributes, using existing accepted standards, when possible, such as the ISO 8601 format for dates, which is largely used, including by SNOMED CT [35] (Figure 2). The advantages of representing scalars directly in formal grammar expressions are vast. This greatly improves querying capacity, using queries with numeric operators, such as “all heart rates with a value of >150 bpm.”

### Negation

Negation is the act of denying or contradicting something, or the expression of its absence or opposite. The ability to represent negation is a key part of any language. Without it, this sentence could not be written. It does, however, pose many challenges when attempting to include it in a knowledge representation, particularly with regard to inference in hierarchical structures [36]. SNOMED CT’s approach to negation through its context model exists outside the DL, as the absence of something is still linked to the focus concept by an “Associated” attribute [37], which is logically incoherent. Other ways of representing negation in SNOMED CT can also lead to errors of inference and classification [36], with children concepts being less restrictive than their parents. In fact, SI goes as far as to recommend “handling negation outside of SNOMED CT ... rather than try and represent it within the terminology” [38].

**Figure 3:**
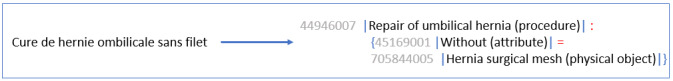
Negation representation example.

However, SNOMED CT abounds with concepts containing negation in some form or another. The limitations described above apply to the approved attribute relations that are used in fully defined concepts. For the cases not already covered, plenty of concepts exist that represent negation in one way or another, such as unapproved attributes and qualifiers, which are used to extend the MRCM when necessary. Extending attribute domains and using new attributes such as “Except for” and “Without” has proven highly useful. For hernia repairs, for example, SNOMED CT only specifies when the procedure is done with a mesh. However, no concepts describe the absence of mesh during a procedure, which was needed (Figure 3). These modifications allow us to cover many instances of negation encountered and are currently deemed sufficient.

### Uncertainty

Uncertainty describes a situation in which something is not known or not certain. This has an important place in representing medical data, as many situations in clinical care contain uncertainty. It is inherent in every patient encounter. It starts with the patient’s history, such as not knowing when a symptom began, and continues through the diagnostic process, from forming a differential diagnosis to choosing which tests to order.

Natively, SNOMED CT represents uncertainty in the same way as negation, with the “Finding context” attribute with values such as “Known possible.” As with negation, however, SI states that “attempts to capture probabilistic or uncertain knowledge are out of the scope of SNOMED CT” [39], despite containing approved attribute relations for describing such situations. It is also present in primitive concept labels such as “Uncertain diagnosis.”

**Figure 4:**
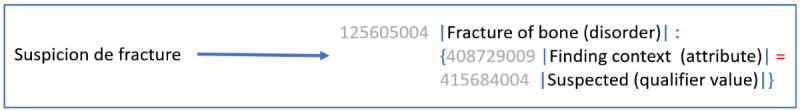
Uncertainty representation example.

Similarly to negation, the extensions made to cover uncertainty are expanding the domain of the “Finding context” and “Procedure context” attributes to include “<<Clinical finding” and “<<Procedure,” respectively. This allows for representing Findings and Procedures with a refinement concerning uncertainty (Figure 4).

### Temporality

Temporality is the state of existing within or having some relationship with time. It is a vital property to consider when analyzing patient data. Indeed, knowing if a certain procedure, laboratory test, or diagnosis happened before, during, or after another is critical and has a defining influence on how information is interpreted. This also includes notions such as duration of processes or procedures, evolution over time, and chronicity.

SNOMED CT already has an extensive coverage of temporality and has proven capable of adequately representing many cases encountered in practice, such as “Temporally related to” (with its descendants After, During, and Before). These apply to various domains such as Situations, Clinical findings, and Observables entities, and therefore already cover many situations. However, despite their extensive coverage, gaps remain in the representation of temporality.

**Figure 5:**
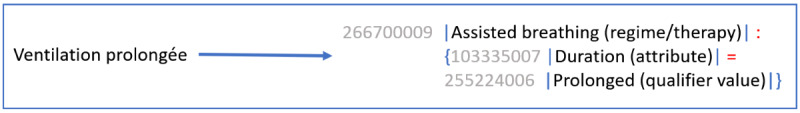
Temporality representation example.

Extensions include extending the range of the Before and During attributes to match that of After. Procedures are added to these attributes’ domains. Some larger gaps include dates, which are resolved using the “Date” attribute, and duration of procedures, with the “Duration” attribute (Figure 5), which both use scalar values as necessary.

### External Vocabularies Integration

#### Overview

While the situations mentioned up to now are certainly a welcome improvement in terms of scope of representation, they describe modifications compliant with SNOMED CT extension capabilities. Other types of encountered gaps, which concern specific group of concepts absent from SNOMED CT, are not resolvable in this way. Representing these concepts, therefore, necessitates the inclusion of external terminologies. While using different standards together can hinder interoperability, as it can limit the possible exchange and secondary usage of data, it has become unavoidable. Integrating various formats and standards to make them compatible has been identified as a challenge in the field of medical informatics [40]. In fact, SNOMED CT itself was born from the fusion of SNOMED RT and Clinical Terms V3, so what is proposed here is only an extension of its original philosophy [41]. However, it should be remembered that multiple maps exist between SNOMED CT and terminologies such as ICD-10 and Orphanet [42], as well as the effort to harmonize LOINC and SNOMED CT [43,44]. These should be consulted first to ensure that the desired term does not already have a SNOMED CT equivalent. Ontology alignment efforts and thesauri such as Unified Medical Language System should also be consulted to verify equivalences.

Once this verification is done, external vocabularies can be evaluated for integration. How they are evaluated is subject to rules. We have defined five selection criteria: (1) the terminology must first cover an identified gap in SNOMED CT coverage; (2) it must be concept-based, so cannot be a thesaurus; (3) it must be hierarchical and contain at least some graph-like properties; (4) it must have versioning capabilities; and (5) it must be a well-recognized standard widely accepted by relevant users. Once chosen, the steps described below should be followed to fully integrate them, increasing the scope of representation. This includes adding them virtually to a SNOMED CT hierarchy by attributing a parent, verifying MRCM rules, and modifying the ABNF syntax.

#### Content Analysis and Proximal Primitive Parent Attribution

**Figure 6:**
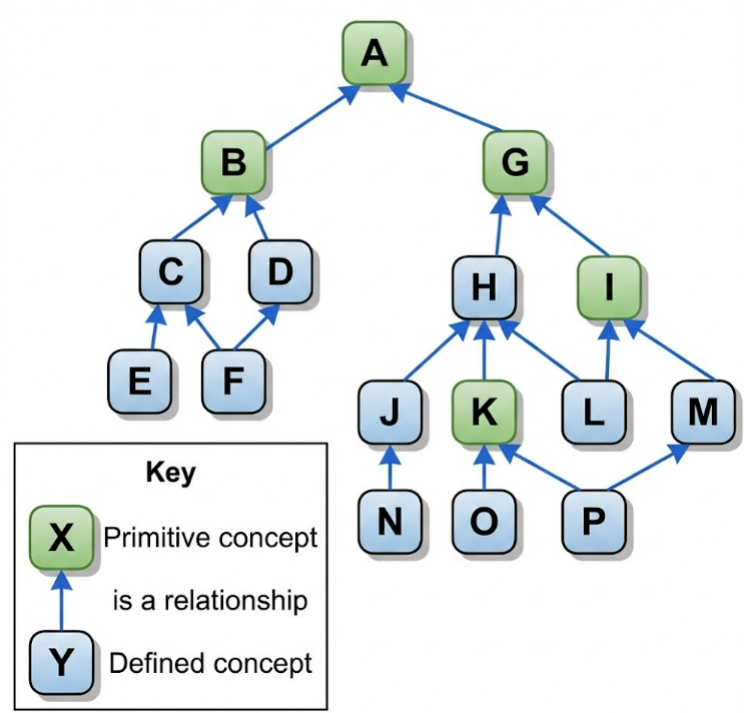
Proximal primitive parent attribution.

To include an external terminology, it is necessary to clearly identify when and where it would be used. The content of the identified terminology should be analyzed in parallel with SNOMED CT to define where it could fit in the SNOMED CT hierarchies. Ideally, an entire terminology should be linked to one parent concept, but if different parts of the terminology fit in different hierarchies, this should be documented, and clusters of codes should be defined if needed. The defined clusters are then attributed to a proximal primitive parent. This task is described in the SNOMED CT authoring course for concept creation and consists of defining the closest parent of the cluster of codes in SNOMED CT that is not fully defined [45]. To do so, the closest parent is assigned, and its genealogy is reviewed to find the first primitive concept (Figure 6 [46]).

#### Modifying the MRCM and ABNF

Once the clusters of codes are attributed to a parent, MRCM rules covering those parents must be reviewed to ensure they are still valid. If necessary, they can then be updated, or new rules created, to include the cluster in the range or domain, if they fit an approved definition. This helps in keeping coherence when creating new PCEs, but must be done carefully, as integrations that happen too high in the hierarchy can influence multiple existing MRCM rules.

A syntax modification is then necessary to allow for integration of external concepts into SNOMED CT PCEs. The ABNF syntax is therefore modified to signal when another terminology is used and to allow validation of PCEs. The tilde symbol (~) is used to signal the start and end of the use of external concepts. Another ~ is used to separate the identifier of the vocabulary and the concept used. These new terminology concepts can, in theory, be inserted into any part of an expression, whether as the focus concept (~Terminology~id~:SCT=SCT), attribute (SCT:~Terminology~id~=SCT), value (SCT:SCT=~Terminology~id~), a combination of these, or even used alone. This modification has been validated using a modified ABNF parser on newly created expressions. The modified ABNF syntax is available in Multimedia Appendix 2.

#### Implementation

**Figure 7:**
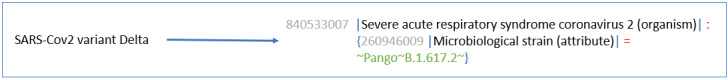
External terminology representation example.

When the COVID-19 pandemic hit, genomic sequencing quickly led to a characterization of the SARS-CoV-2 virus in specific strains, with scientific and vernacular names given by organizations such as Nextstrain [47], Pango [48], and the World Health Organization [49]. These strains took on a crucial importance with the advent of more contagious or less lethal variants such as Delta or Omicron [50]. This close focus on specific variants is well represented in classifications such as the Pango nomenclature [51]. However, in SNOMED CT, the representation of those strains is lacking, with a unique code to characterize the SARS-CoV-2 virus. This situation is used as a use case to demonstrate the inclusion of an external vocabulary through the proposed method. In this scenario, Pango is chosen to refine the organism hierarchy for SARS-CoV-2 lineages. Therefore, a new attribute group with a domain, range, and definition must be validated. Since SNOMED CT already has a category of codes that defines strains but does not include SARS-CoV-2, the Pango nomenclature is added as children of this code. A new MRCM rule is then defined to refine the Organism hierarchy with the “Microbiological strain” attribute, as shown in Figure 7.

Using this method, a total of 5 terminologies other than SNOMED CT are added to expand the scope of representation. These are Pango, Nextstrain, Sequence Ontology [52], RadLex [53], and the Common Terminology Criteria for Adverse Events (CTCAE) [54].

## Discussion

### Principal Results

#### Overview

This work addresses the difficult balance between expressivity and standardization with a first step in the direction of an FDG for semantic representation of clinical data, using SNOMED CT as the base. It provides a formal, extensible set of rules for constructing meaning that can draw from diverse vocabularies as needed. This modular approach means new terminologies can be integrated as they become relevant without limiting what can be said, but providing a clear structure for how to say it.

After 10 years of representing structured data in the HUG datalake, the FDG now provides semantic representations for 119,941 distinct data elements using 39,168 unique expressions. These expressions cover over 13 billion data instances from 1.7 million patients. Notably, 18,370 of these unique expressions are PCEs, validating the approach as nearly half of all unique expressions created are complex PCEs. This clearly demonstrates that precoordinated concepts alone are profoundly insufficient to capture clinical reality at scale. Moreover, 4500 PCEs use the extensions presented in this paper, for a total of nearly 500 distinct extended PCEs, underscoring their usefulness.

This approach differs from existing interoperability efforts. While FHIR provides a flexible structural framework to define custom data models (FHIR profiles), its focus is not on prescribing semantic content, leaving users to define their own semantic frameworks. In comparison to FHIR extensions, the approach is similar, but we connect the external terminology to SNOMED CT to maintain semantic coherence. To the best of our knowledge, this is not allowed in FHIR extensions, which do not specify the relation of the extension to the resource it extends. Crucially, neither FHIR nor OMOP supports the definition of PCEs, a capability central to our solution for expressing complex clinical nuances. In comparison to ontology alignment frameworks, our approach does not align ontologies but allows their combined use to compose concepts absent in both. Furthermore, a key difference lies in the methodology; our solution focuses entirely on the semantic description of data where it resides, avoiding the necessity of data movement or physical transformation inherent in many FHIR or OMOP-based implementations. Because our primary intent is semantic description and not structural or physical data modeling, a direct, formal comparison between these solutions is not appropriate.

The decision to build upon and extend SNOMED CT is grounded in the need for a robust and logical foundation, which its unique extensible grammar and syntax provide. This work shows that extending a powerful existing standard can be a path toward semantic interoperability. Four large domains of clinical reality, identified as lacking in SNOMED CT and known as challenging fields to represent, have been partially solved using this approach. Despite allowing for the representation of a large amount of data elements that were out of reach of current SNOMED CT rules, there remain gaps in representation that have not yet been resolved. Each category described previously has seen its coverage improved, but as things stand, complete coverage of these domains is still out of reach.

A statistical analysis of the extensions used shows that the 4500 PCEs created using extensions represent less than 4% of data elements and 3% of instances (Table 1). However, a deeper analysis shows that while seemingly sparse overall, extensions are very useful for certain data types. Laboratory procedures and medical devices, for example, are two collections in which 27% (250,617,656/935,678,204) and 23% (1,140,820/4,926,815) of instances, respectively, were represented with extensions. Other collections, such as formularies, have nearly 4000 extensions used.

**Table 1:** Detailed statistics of representation by category of data using extensions.

Source	IDs encoded, n	Extensions used, n (%)	Instances encoded	Extension instances, n (%)
Administrative stay	1172	2 (0.17)	78,020,550	131,832 (.17)
Anesthesiology	2139	11 (0.51)	117,419,522	1,275,337 (1.09)
Medical devices	699	235 (33.62)	4,926,815	1,140,820 (23.16)
Formularies	51,322	3925 (7.65)	1,641,633,208	119,941,273 (7.31)
ICU^a^	6934	157 (2.26)	6,656,119,500	367,548 (.005)
Laboratory	7231	72 (1)	935,678,204	250,617,656 (26.78)
Observations	2784	1 (0.03)	2,440,891,370	1003 (.00004)
Patient problems	25,233	72 (0.28)	1,587,507	976 (.06)
Prescription	9837	0 (0)	26,805,381	0 (0)
Radiology	2586	2 (0.07)	8,562,371	2 (.00002)
Procedures	8001	23 (0.28)	1,452,038,108	8,934,163 (.6)
Total	119,941	4500 (3.75)	13,362,644,974	382,410,610 (2.86)

^a^ICU: intensive care unit.

Closer examination shows clearly that the extensions have proven extremely useful to fill certain specific gaps within SNOMED CT. In these cases, the gaps identified were SARS-CoV-2 strains for laboratory procedures, sizes for medical devices, and occupations for formularies. Looking in even further detail, the 4500 PCEs created represent 488 distinct expressions. However, certain specific expressions have proven very useful. Extending representation of negation was a big part, with the Without and Except for attributes used a combined 88 times, and sizes for medical devices used 178 times.

#### Machine-Readable Interoperability

A primary concern regarding the modification of established standards is the potential loss of machine readability. Our implementation presents a dichotomy in this regard. The modifications made strictly to the MRCM, such as domain and range extensions for scalars or negation attributes, remain compliant with SNOMED CT’s structure. These are natively processable by standard terminology servers like Snowstorm, provided the modified MRCM is loaded [55].

However, the integration of external vocabularies required modifications to the ABNF syntax (specifically the use of the tilde delimiter), which constitutes a divergence from the standard CG. Consequently, standard parsers and expression constraint language query engines cannot currently process these specific expressions without adaptation. We have validated that these expressions can be parsed using a modified ABNF parser, and we are currently adapting open-source tools to accommodate this syntax. Until these tools are widely available, this specific aspect of the grammar hinders immediate interoperability with the existing global standards ecosystem.

This technical gap is compounded by a governance challenge, as the number of integrated terminologies increases, so does the need for robust documentation to prevent ambiguity. A formal glossary or registry is therefore required to track which terminologies are in use, their specific versions, and the rules governing their application. For clarity, versioning could even be embedded directly within an expression, for example: ~CTCAE~v5.0~4028512~. A more sustainable formalism could use Uniform Resource Identifiers to designate terminology releases. This could be implemented without modifying the ABNF.

#### Challenges Encountered

Scalars are a massive chapter, and representing certain situations, such as ranges, has not been resolved yet. Allowing for scalar values in the range of most hierarchies also requires defining new attribute relations for each use case encountered. Negation is resolved by exclusively using and extending existing SNOMED CT concepts, as this is sufficient for our needs and maintains internal consistency within the grammar. However, this is not the only valid approach. More complex scenarios could be addressed by incorporating a formal negation operator, such as the widely accepted “¬” symbol, as described by Schulz et al [37]. Similarly, reification offers another method for handling negation within SNOMED CT, though it lacks a directly queryable negation indicator [36,37]. This would require additional ABNF syntax modification, and the impact it would have on DL means that this has not been implemented.

Other challenging situations fall into different categories but account for only a small fraction of all data elements, such as rare, complex labels within otherwise well-resolved categories. These include highly specific laboratory procedures that represent less than 1% (1,475,290/250,521,754) of all laboratory procedure instances. They could likely be addressed by incorporating more specialized terminologies. However, because these cases are anecdotal, the effort required to search for and integrate additional terminology is not justified for now.

Data-element categories with no widely adopted standard and no suitable SNOMED CT codes for creating new PCEs are another challenge. An example is triage-sheet questions, which include 350 distinct labels across 4 million instances, a negligible portion of the overall dataset. Work is ongoing to find solutions, primarily through new MRCM rules, such as defining an appropriate relationship between Observable Entities and Clinical Findings (current options like “Precondition” or “Has realization” are inadequate), or between Environments and Procedures.

Finally, some grammatical constructions are poorly supported by current grammar rules. A key case is the presence of “or” in problem-list entries. For concepts containing “or,” we currently apply a rule that the label is represented only if both concepts share a sufficiently close semantic parent; otherwise, it remains unmapped. Some labels can be resolved this way, such as Intervention de gynécologie ou d’obstétrique = Operation on female genital organs (procedure). But others are too semantically broad or distant, with no precise match, such as Pneumonie ou pneumopathie, where the shared parent Disorder of lung (disorder) is considered too vague and would cause excessive information loss. In total, our database contains 2895 labels with “or,” representing 17.5 million instances. Among these, 834 (28%) labels have been successfully resolved, covering 10 (57%) million instances.

### Limitations

This work presents limitations. For reasons mentioned previously, no interannotator agreement was carried out on the work done. The goal was initially to develop the approach for internal use only. Since it evolved gradually in parallel to our improving capabilities, there was never a “before” and “after” to evaluate. Also, since the team is made up of a set core of 3-4 members, it was considered more useful for us to evaluate our work in focus group discussions. There was never a need internally for a more explicit evaluation because problems are reviewed so often. Finally, while the intention is to generalize the way people use SNOMED CT to represent data, one of the founding principles of SNOMED CT is that there are many ways to express the same thing. As the most important outcome is semantic accuracy, maintaining the meaning of the data element, if two expressions mean the same thing but are written differently, they are still both correct. The expected outcomes were therefore never exactly matched. Such subtleties would not appear in a formal interannotator agreement, reducing its interpretability, an important point, which, in our opinion, diminished the potential impact of such an evaluation.

The byproduct of an inclusive grammar, as described in this work, is that it will inevitably make reuse more difficult. There will be links created that should not work, and inconsistencies in DL. The primary limitation of this implementation lies in the operational integration of external vocabularies with standard SNOMED CT tools. While separation of grammar and vocabulary was identified as a crucial need for an FDG, its implementation in the proposed approach is incomplete. The MRCM we chose to keep and extend constitutes a set of rules that are semantic, not merely grammatical. Currently, the terminologies added to the grammar are attributed a SNOMED CT code as a parent, to allow initial querying. But the new codes themselves cannot be directly accessed yet. For this to function, the integrated terminologies need to be definitively added to SNOMED CT. There are ways to do this in theory, such as creating a new concept with a valid SNOMED CT concept identifier for each, but they have not yet been explored in detail or implemented. Applying these solutions would greatly enhance reuse capabilities, improving interoperability and consistency of results. Modifying the ABNF syntax and parser is a first step in this direction.

The results only cover the sources that have been selected for representation so far. As the project continues, new sources will be analyzed, extracted, and represented. Therefore, it is not possible, to date, to give a clear image of the progress compared to the complete data warehouse. However, the choice of the sources to be added first has been designed to cover first the core of the electronic health record, before smaller sources. Additionally, new problems will inevitably appear, which will need to be addressed, and new rules will have to be added. As this work focuses primarily on structured data, it is not possible to confront our semantic coverage to the full information content of the electronic health record, but this is being addressed through automatic annotation of free text using natural language processing in a similar manner.

Finally, the approach was tested only in our environment and is currently used nowhere else. The end goal, however, is, of course, to apply it elsewhere to test its reproducibility, as the complexity and variability of the approach mean it may not be immediately applicable in different settings.

### Governance and Implementation

Key recommendations can be derived from this work to address the limitations discussed above. To safely adopt these extensions, institutions should establish a minimum governance structure consisting of a multidisciplinary committee of domain experts and SNOMED CT–certified terminologists. This body acts as a gatekeeper, validating new representation rules through a consensus-based cycle only when standard SNOMED CT concepts or official maps (eg, LOINC and Orphanet) are proven insufficient. Key steps toward alignment with SI include maintaining a formal local registry of all grammar extensions and prioritizing the use of standard extension mechanisms (MRCM) over syntax modifications to preserve compatibility with the broader ecosystem. In terms of maturity, the MRCM extensions for scalar values, temporality, uncertainty, and negation are stable and ready for immediate reuse in standard terminology servers (eg, Snowstorm). Conversely, the ABNF syntax modifications required for integrating external vocabularies remain experimental; they currently necessitate custom parsing tools and can be adopted as a proof-of-concept pending the evolution of standard tooling.

### Future work

Steps have been taken to start implementing the strategy in different institutions, as interest has been strong, and collaborations are currently ongoing, but are at a preliminary stage for the moment. External replication and independent validation are being undertaken, which aim to evaluate the adaptability and reusability of our approach, demonstrating its generalizability. Additionally, specific projects in the hospital have already benefited from using our approach to gather data on subjects such as biomarkers in the field of genomics, which was not possible before.

Furthermore, while this work relied on expert manual representation, future research will focus on automating the translation of free-text clinical notes into PCEs using natural language processing. Emerging methodologies leverage large language models (LLMs) equipped with retrieval-augmented generation to ground generated expressions in the extensive SNOMED CT hierarchy, a technique showing potential for high-fidelity clinical coding [56]. However, we acknowledge that the generation of structured sequences via LLMs introduces specific risks, such as exposure bias, which can affect the reliability of the output. Recent work highlights the importance of mitigating these biases in LLM distillation to ensure robust structured generation [57]. Addressing these challenges would dramatically scale implementation and unlock the potential for advanced semantic reasoners to infer new knowledge from grammatically rich, machine-readable clinical data.

### Conclusions

This methodological study demonstrates how SNOMED CT’s CG can be leveraged and extended to address recurrent semantic gaps encountered in a large-scale clinical data warehouse. Rather than constructing a new FDG from scratch, we have approached this theoretical goal by systematically extending the capabilities of an existing standard. Through specific modifications to the MRCM and syntax, we successfully addressed complex representational challenges such as negation, scalar values, and the integration of external terminologies, thereby reducing the tension between clinical expressivity and standardization.

Supported by a decade of implementation at HUG, this work illustrates that a grammatical framework, separating the rules of composition from the vocabulary itself, is essential for capturing meaning at scale. While standardized vocabularies provide the necessary lexical building blocks, it is the flexibility of the grammar that determines the fidelity of the representation. This study serves as a proof-of-concept that semantic interoperability can be advanced by methodically extending the expressive power of existing standards. However, the widespread adoption of such extensions requires robust governance frameworks to collaboratively manage and share grammatical rules across institutions.

## Appendix

Multimedia Appendix 1 Machine Readable Component Model modifications and additions.

Multimedia Appendix 2 Modified Augmented Backus-Naur Form syntax.

## Abbreviations

ABNF: Augmented Backus-Naur Form
CG: compositional grammar
CTCAE: Common Terminology Criteria for Adverse Events
DL: Description Logics
FDG: formal descriptive grammar
FHIR: Fast Healthcare Interoperability Resources
HUG: Geneva University Hospitals
ICD-10: International Statistical Classification of Diseases, Tenth Revision
ISO: International Organization for Standardization
LLM: large language model
LOINC: Logical Observation Identifiers Names and Codes
MRCM: Machine Readable Component Model
OMOP: Observational Medical Outcomes Partnership
PCE: postcoordinated expression
SI: SNOMED International
SNOMED CT: Systematized Nomenclature of Medicine – Clinical Terms
